# Silica–Ti_3_C_2_T_x_ MXene Nanoarchitectures with Simultaneous Adsorption and Photothermal Properties

**DOI:** 10.3390/ma17174273

**Published:** 2024-08-29

**Authors:** Eduardo Ruiz-Hitzky, Mabrouka Ounis, Mohamed Kadri Younes, Javier Pérez-Carvajal

**Affiliations:** 1 Materials Science Institute of Madrid (ICMM-CSIC), c/Sor Juana Inés de la Cruz 3, 28049 Madrid, Spain; mabrouka.ounis@etudiant-fst.utm.tn (M.O.); jperez@icmm.csic.es (J.P.-C.); 2 Laboratory of Materials Chemistry and Catalysis, Department of Chemistry, Faculty of Sciences of Tunis, University of Tunis El Manar, Tunis 2092, Tunisia; younes.mohamedkadri@gmail.com

**Keywords:** MXenes, Ti_3_C_2_T_x_, 2D solids, silica, nanoarchitectures, photothermal materials, intercalation, supported reactions

## Abstract

Layered Ti_3_C_2_T_x_ MXene has been successfully intercalated and exfoliated with the simultaneous generation of a 3D silica network by treating its cationic surfactant intercalation compound (MXene-CTAB) with an alkoxysilane (TMOS), resulting in a MXene–silica nanoarchitecture, which has high porosity and specific surface area, together with the intrinsic properties of MXene (e.g., photothermal response). The ability of these innovative MXene silica materials to induce thermal activation reactions of previously adsorbed compounds is demonstrated here using NIR laser irradiation. For this purpose, the pinacol rearrangement reaction has been selected as a first model example, testing the effectiveness of NIR laser-assisted photothermal irradiation in these processes. This work shows that Ti_3_C_2_T_x_-based nanoarchitectures open new avenues for applications that rely on the combined properties inherent to their integrated nanocomponents, which could be extended to the broader MXene family.

## 1. Introduction

MXenes are a relatively recent, broad family of inorganic solids discovered at Drexel University [1] consisting of two-dimensional (2D) transition-metal carbides, nitrides, and carbonitrides of the general formula M_n+1_X_n_ (n = 1–3), where “M” represents transition metals (e.g., Ti, Zr, Ta, Nb, V, Mo, Cr, etc.) and “X” is carbon and/or nitrogen elements. MXenes are obtained from the so-called “MAX” phases (layered ternary carbides and nitrides with a general formula M_n+1_AX_n_, where “A” represents elements from groups 13 and 14 of the periodic table). The etching of these A-elements generates a large family of two-dimensional carbides and nitrides (MXenes) endowed with attractive physicochemical properties that combine excellent hydrophilicity and high electrical conductivity. Another important feature of MXenes is their high photothermal conversion efficiency, which allows for light-to-heat conversion, useful for the photothermal applications of their corresponding derivatives. In addition, they show a large redox active surface area, rich surface chemistry functionality, and outstanding mechanical properties, and, as occurs with many 2D solids, exhibit intercalation ability [1,2,3,4,5,6,7,8].

The large family of MXenes constitutes an important group of sustainable and even biocompatible materials that are nowadays considered promising candidates to replace graphene and other carbon-based 2D solids. MXenes are currently being thoroughly investigated, from their preparation to their thermal and chemical stability and for their uses in a wide range of applications, including energy storage and conversion, electronics, sensor devices, biomedicine, environment preservation, and catalysis [9,10,11,12,13]. Besides the impressive electrical conductivity shown, they exhibit some properties comparable to those of to clays and clay minerals, receiving the nickname of “conducting clays” or “carbide clays” [14]. However, the term “clay” should preferably be associated with silicates and related small-size particles present in the geosphere [15]. Based on their intercalation and delamination properties, clay phyllosilicates can nowadays be considered the most representative 2D inorganic solids capable of generating a wide variety of nanoarchitectured materials. This feature is very similar to that described in MXenes and facilitates interactions between the single layers of these solids and various inorganic species (e.g., metal and metal-oxide nanoparticles), as well as with many diverse organic compounds, giving rise to organic–inorganic hybrid materials [15,16].

The controlled arrangement of nanosized structural units based on nanoarchitectonic approaches is resulting in a major boost in the design of advanced functional nanoarchitectures. In this sense, 2D solids with intercalation and delamination properties that could involve surface interactions of their single layers present extraordinary potential as building blocks for these purposes [17]. In this way, layered silicates (clay minerals) and, more recently, MXenes are excellent candidates for generating different functional nanoarchitectures [11,15]. The exfoliation/delamination of 2D solids allows for further conformations as films (restacking) or foams (freeze-drying), which, in addition to their ability to assemble diverse nanoparticles, polymers, or biological fragments, can generate functional nanoarchitectures suitable for a wide range of applications, from the reinforcement of polymeric matrices and membrane technologies to several transport properties, like optical, electronic, magnetic, etc. [18].

The possibility of designing complex systems with individual layers of different 2D solids highly dispersed in silica matrices allowing for the combination of their textural properties (high specific surface area and porosity) with the inherent properties of the 2D solid (e.g., MXene) represents a very attractive opportunity. For example, delaminated clay minerals and related solids, like layered double hydroxides, associated with silica generated by sol–gel processes have been a breakthrough in the production of clay–silica nanocomposites with synergistic properties of both components, i.e., the surface properties of the silica and the ion exchange properties of the involved clay mineral [19,20]. Intermediate phases of 2D solids modified by the intercalation of cationic alkylammonium species (e.g., hexadecyltrimethylammonium bromide, CTAB) have been found to generate an organic–inorganic interface that facilitates the association and stabilization of alkoxysilanes and other alkoxides, providing a suitable environment for the subsequent hydrolysis and controlled polycondensation of the alkoxides to form 3D silica networks [19,20,21]. In addition to alkoxysilanes, Al- and Ti-based alkoxides, as well as other types of metalorganic precursors, have also been successfully used to introduce selected functionalities (acidity, photoactivity, etc.) [21,22,23,24]. Previous systems dealing with the assembly of MXenes and silica have been successfully produced by the co-intercalation of tetraethyl orthosilicate (TEOS) with long chain alkylamines. The resulting materials have been consolidated by calcination, giving rise to Ti_3_C_2_T_x_ MXenes with SiO_2_ pillars, characterized by a permanent increase of the basal interlayer spacing up to 3 nm, as indicated by the corresponding XRD patterns. These materials also show a significant increase in their specific surface area, reaching values greater than 200 m^2^ g^−1^ [25].

**Figure 1:**
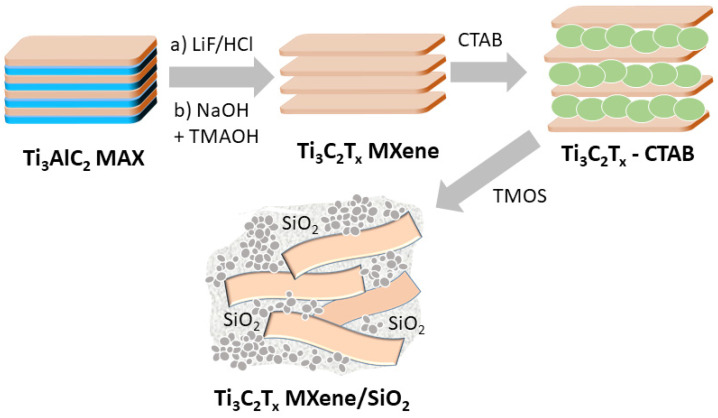
Scheme of the pathway adopted for the synthesis of MXene-SiO_2_ nanoarchitectured materials starting from Ti_3_AlC_2_ carbide (MAX phase) by treatment with LiF in HCl solution to produce Al etching (a) and neutralization/delamination (b), followed by several steps involving the use of the MXene-CTAB intermediate and the TMOS hydrolysis reaction, leading to the MXene-silica nanocomposite.

Herein, we report the synthesis of new nanoarchitectures derived from Ti_3_AlC_2_ carbide (MAX phase) leading to a high dispersion of Ti_3_C_2_T_x_ MXene single layers within a silica framework generated from tetramethyl orthosilicate (TMOS). This procedure involves the preparation of an intermediate phase by the intercalation of MXene, using the cationic surfactant CTAB (MXene-CTAB), which facilitates the exfoliation and subsequent assembly with the SiO_2_ generated by the hydrolysis and polycondensation of TMOS (Figure 1). The final objective of this work is to prepare new nanoarchitectures based on MXene–silica that simultaneously present high porosity and, therefore, adsorption capacity, together with photothermal properties inherent to MXene layers, with the property of activating chemical reactions of the adsorbed species.

## 2. Materials and Methods

Ti_3_AlC_2_ (MAX phase) was acquired as a 200-mesh powder from Shanghai Epoch Material Co., Shanghai, China). Chemical reagents provided by Sigma-Aldrich Merck KGaA, Darmstadt, Germany, were used without further purification: tetramethyl orthosilicate (TMOS) and LiF (analytical reagents grade), NaOH (97% purity, pellets), hydrochloric acid (37% wt. HCl), cetyltrimethylammonium bromide (CTAB) (≥98%), and tetramethylammonium hydroxide (TMAOH) (25 wt. % in H_2_O). Sulfuric acid (95–97%, Merck, Darmstadt, Germany), pinacol (98%, Aldrich, Steinheim, Germany), and 2,4 dinitrophenylhidrazine (*purum* quality) were provided by FEROSA.

### 2.1. Synthesis of the Ti_3_C_2_T_x_ MXene

In a PTFE vial, 0.80 g of LiF was dissolved in 10 mL of 9 M HCl under continuous stirring, and then 0.25 g of Ti_3_AlC_2_ (MAX) was gradually added to the mixed solution and reacted at room temperature for 72 h. Then, the acidic product was neutralized using 11 M NaOH until pH ≥ 7, incorporating also 4 mL TMAOH as an etchant agent. The obtained product was then repeatedly washed with deionized water and methanol with centrifugation at 3500 rpm. Finally, after more than one hour of continuous centrifugation, the resulting Ti_3_C_2_T_x_ MXene was collected from a concentrated dark green supernatant solution.

### 2.2. Synthesis of MXene-CTAB and MXene-CTAB-SiO_2_

A total of 100 mg of Ti_3_C_2_T_x_ MXene was dispersed in 10 mL of deionized water and added to 10 mL of 7.08 mM cetyltrimethylammonium bromide (CTAB) solution under stirring for 3 days at room temperature. The suspension was then centrifuged at 3500 rpm, and the precipitate was dried at 60 °C overnight to obtain the MXene-CTAB sample. For the MXene-CTAB-SiO_2_ preparation, the acquired MXene-CTAB was homogeneously dispersed in isopropyl alcohol. Then, an equimolar amount of the alkoxysilane (1:1 MXene/silane) was slowly added to the suspension under continuously stirring for 10 min. Subsequently, water and methanol were added dropwise to the system with a molar ratio of TMOS/H_2_O/methanol of 1:2:2. This mixture was kept under magnetic stirring for 72 h at room temperature, then centrifuged and dried. Finally, the extraction of CTAB from MXene-CTAB-SiO_2_ was performed in ethanol solution containing 0.1 M HCl at 70 °C for 24 h. The resulting dried sample was named MXene-SiO_2_.

### 2.3. Characterization Techniques

Powder X-ray diffraction (XRD) diagrams were collected in a Davinci instrument from BRUKER in the range between 4 and 70° (2ϴ) angles. FE-SEM images were obtained using a FEI NOVA NANOSEM 230 equipped with an EDAX-Ametek detector (Eindhoven, The Netherlands). TEM images and EDX were obtained with a JEOL JEM-1400 Plus electron microscope (Tokyo, Japan). Nitrogen adsorption isotherms at 77 K were determined in a Micromeritics (Norcross, GA, USA) 3Flex instrument after outgassing the solid powder at 120 °C during 12 h under dynamic vacuum. Attenuated total reflection Fourier transform infrared spectroscopy (ATR-FTIR) was performed using an ALPHA II spectrometer (Bruker Corporation, Billerica, MA, USA). The spectra were acquired between 500 and 4000 cm^−1^ with a sample scan time of 256 scans and a resolution of 4 cm^−1^. Five replicates were acquired for each sample.

### 2.4. Photothermal Properties

To investigate the photothermal properties of the prepared MXene and MXene derivatives, they were irradiated with 808 nm wavelength laser light (830 mW). The temperature was continuously recorded for 2 min; then, the light source was switched off until the temperature decreased to room temperature. Throughout the irradiation process, thermal images and temperatures changes in the materials were captured with an infrared thermal camera (HIKMICRO, Hangzhou, Zhejiang, China).

The photothermal activation of the pinacol transposition reaction to pinacolone was carried out by dropping a saturated pinacol solution on to H_2_SO_4_-doped MXene-SiO_2_. Then, the sample was exposed to an attenuated NIR laser to heat a pressed millimetric disc of powder reaching ca. 80 °C during 10 min. Thin discs of the powdered product were shaped by applying 4 tons/cm^2^ for 10–20 s in a mechanical press. The detection of the formation of carbonyl compounds from pinacol was performed qualitatively by dropping 2,4-dinitrophenylhydrazine (2,4-DNP) into the isopropanol extracted phase. The formation of a yellow-orange precipitate confirmed the presence of a carbonyl group (C=O) in the pinacolone molecule.

## 3. Results

The general procedure followed here to prepare the MXene–silica nanoarchitectured materials (Figure 1) is inspired by our previously developed protocol on the preparation of clay–silica nanocomposites [21]. When this procedure is applied to MXenes, the first step concerns the preparation of Ti_3_C_2_T_x_ MXene from the MAX phase (Ti_3_AlC_2_). It consists in the treatment of this solid with LiF dissolved in concentrated HCl that produces the etching of the Al layer of MAX, as reported by Ghidiu and co-workers [14]. The resulting products are then neutralized with dilute NaOH, also incorporating tetramethylammonium hydroxide (TMAOH), which can act as both a neutralizer and an etchant agent. The amphoteric nature of Al^3+^ allows for its extraction from the MAX structure, giving rise to MXenes with Al(OH)_4_^−^ species as terminal groups [26]. In the second step, the cationic surfactant (CTAB) is intercalated in the resulting fresh MXene with the aim of expanding its interlayer spacing to facilitate the further interlamellar access of the TMOS alkoxide used as a silica source. Once MXene-CTAB-SiO_2_ nanocomposites are generated, the extraction of the surfactant is achieved by treatment with a mixture of ethanol/HCl solutions, which give rise to the targeted MXene-SiO_2_ nanoarchitectures.

**Figure 2:**
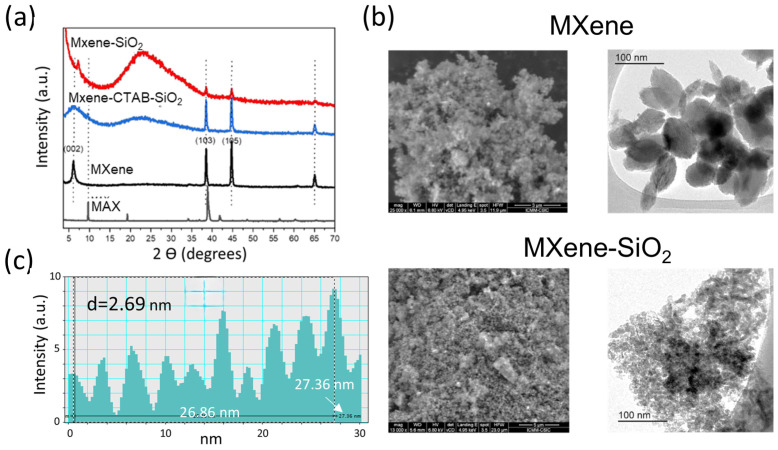
(**a**) XRD patterns of Ti_3_C_2_T_x_ MAX phase, Ti_3_C_2_T_x_ MXene resulting from the MAX phase by reaction with LiF/HCl solution, MXene-CTAB-SiO_2_ intermediate compound, and MXene-SiO_2_ nanoarchitecture after removal of CTA^+^ species; (**b**) FE-SEM (**left columns**) and TEM images (**right columns**) of MXene and MXene-SiO_2_, respectively; (**c**) line profile corresponding to the MXene sample resulting from graphical treatments of the TEM image.

The powder X-ray diffraction (PXRD) patterns (Figure 2a) confirm the formation of the Ti_3_C_2_T_x_ MXene from the MAX phase by reaction with LiF/HCl solution [14]. The characteristic diffraction peak assigned to (002) plane is broader and has shifted to lower angles in comparison to the one present at the MAX phase, indicating an increase in the interlayer distance (d_L_) from 0.91 to 1.45 nm. This fact points to the removal of the Al layer from Ti_3_AlC_2_, which we subsequently confirmed by EDX analyses (*vide infra*). In addition to plane (002), planes (103) and (105) belonging to the layered carbide structure are clearly identified in the resulting material, corresponding to 0.23 and 0.20 nm, respectively. A similar profile has recently been reported by Wang and co-workers [27], interpreting such a diffractogram as indicative of an incomplete etching of the MAX phase based on the presence of remaining peaks assigned to (103) and (105) planes. However, the energy-dispersive X-ray spectroscopy (EDX) results (Figures S1 and S2, ESI) show a low Al content (≤2 wt. %) in the etched MAX sample (Figure S3, ESI), proving the extraction of the majority Al layer. The elemental composition of the prepared MXenes also shows the presence of trace amounts of Cl (2.3 wt. %) and the existence of a large amount of F (9.8 wt. %) and O (25.1 wt. %), suggesting that the terminal surface groups (T_x_) are mainly –O and –F terminations. The observed increase in basal spacing after Al extraction from the MAX phase can be attributed to intercalation between the MXene sheets of water molecules and cations, i.e., Li^+^ from LiF, Na^+^, and TMA^+^ from the corresponding hydroxides used for controlling the pH dispersion [14,28]. In addition, methanol molecules could co-intercalate with H_2_O, playing an active role in the subsequent delamination/exfoliation process and facilitating the access of reagents to the interlayer region.

Treatment with CTAB and TMOS leads to an MXene-CTAB-SiO_2_ intermediate nanocomposite where silica nanoparticles are formed. The diffraction peak assigned to the (002) plane widens further, while a broad hump appears centred at approximately 22° (2θ degrees). This type of diffraction pattern is characteristic of the amorphous silica produced by the hydrolysis of the alkoxides (TMOS), in accordance with the Standards Joint Committee on Powder Diffraction (JCPDS), corresponding to the standard pattern of amorphous SiO_2_ [29]. The presence of CTAB is evidenced from the ATR spectra, showing characteristic peaks at 2922, 2850, and 1460 cm^–1^ that are, respectively, assigned to the asymmetric and symmetric stretching vibrations of C–H (ν_asym_(-CH_2_) and νs(-CH_2_)) and δ (-CH_2_) bending vibrations of methylene groups in the CTA^+^ species assembled to the MXene. The intense IR bands at around 1060 cm^−1^ are attributed to the ν_Si-O_ stretching vibrations of silica siloxane bonds (Figure S4, ESI).

The MXene-SiO_2_ nanoarchitecture is finally produced after the removal of CTAB, included as CTA^+^ cations in the MXene-CTAB-SiO_2_ sample, which is achieved by treatments with an ethanol/HCl mixture. The XRD pattern (Figure 2a) shows a broad band centred at 22° (2θ), attributed to amorphous silica along with a broad peak at 6.08° (2θ) corresponding to the (002) plane of the layered carbide. The latter signal appears here with a relatively small intensity compared to the intensity of the other observed reflexions of the carbide (i.e., the (103) and (105) planes). The broadness of the peak corresponding to the (002) plane can be attributed to the distinct interlayer distances produced by the silica access to the MXene interlamellar spacings.

The resulting spacings vary over a wide range, and the MXene layers are poorly ordered, resulting in structural disorganization in the stacking of the carbide single layers. Figure 2b shows representative FE-SEM and TEM images of MXene-SiO_2_ samples. The FE-SEM/EDX results of the MXene-derived nanoarchitectures show the dispersion of the layered carbide among silica particles with small sizes, typically less than 10 nm. At higher magnifications, it is observed that the latter nanoparticles are closely associated with the MXene, and, in good agreement with the XRD results, the 2D carbide should be present as a few-layers MXene, or as a disordered/delaminated solid dispersed on the generated silica matrix. TEM images show a certain organization in the stacking of the MXene layers, which appears as a nanostructured solid with an almost regular 00l arrangement that periodically repeats. Figure 2c shows the line profile derived from the TEM image that could be related to the d-spacings of 2D solids [30], indicating an estimated average distance between consecutive planes of d = 2.7 nm. This expansion is attributed to the interlamellar insertion of silica in situ, generated by the hydrolysis of TMOS in an environment of the MXene layers with CTA^+^ cations as intercalated species. Semi-quantitative EDX analytical results point out a relatively high content of Ti and Si elements (belonging to carbide and silica, respectively) as well as a low content of Al compared to the starting MAX phase. An appreciable amount of O and F content is also detected, the former being associated to the assembled silica, and the latter consisting mainly of -F terminations. Elements such as Al, Ca, etc., which could contribute to insolubilize fluorides, are not detected, supporting the assignation of –F as T_x_ terminations in the MXene layers (Figure S2, ESI).

The d-spacing value measured from the line profile deduced from TEM images should be taken with caution since it is not the result of conventional measurements using diffraction techniques, but it represents an approximation that is consistent with similar results obtained from related previously prepared MXene–silica systems [25]. It should also be considered that a partial MXene exfoliation or delamination is also produced, whose identification by TEM in the silica matrix would be very difficult to observe due to the very thin single-layer thickness of the carbide and considering its folding ability, which leads to corrugated/rolled solids. The effectiveness of the CTAB extraction to produce the nanoarchitecture is confirmed by ATR spectroscopy, as it shows the absence of C-H vibrations typical of the cationic surfactant (Figure S4, ESI) which was detected in the intermediate MXene-CTAB sample.

**Figure 3:**
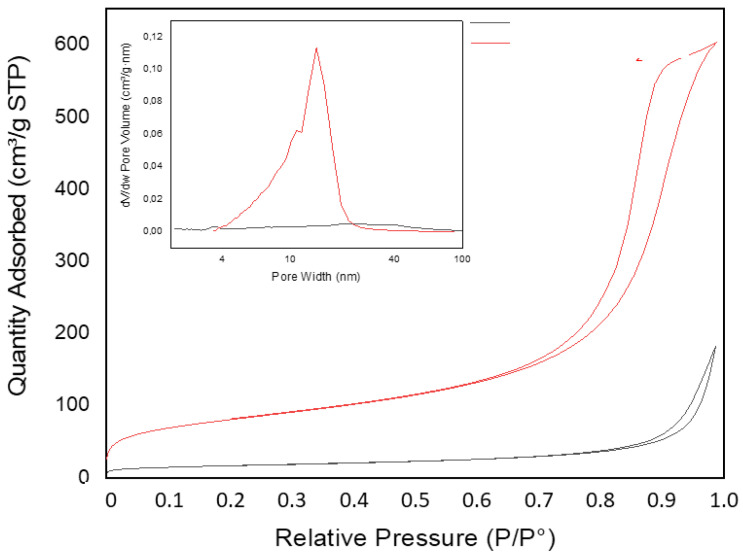
Nitrogen adsorption isotherm and pore size distribution at 77 K of MXene (black) and MXene-SiO_2_ (red).

The resulting MXene-SiO_2_ nanoarchitectures show a significant increase in porosity and specific surface area with respect to the pristine MXene, showing the development of new pores in the range of mesopores (Figure 3). The new porosity developed here is in good agreement with the previously reported development of porosity by the aggregation and growth of silica nanoparticles onto delaminated 2D materials, like smectite clay minerals and vermiculites [19]. The adsorption of N_2_ at liquid nitrogen temperature on MXene-SiO_2_ nanoarchitectures shows type-IV, H1-type isotherms associated with porous materials consisting of well-defined cylindrical-like pore channels or agglomerates of compacts quasi-uniform spheres [31]. Meanwhile, the pristine MXene can be ascribed to type-III, H3-type, ascribed to plate-like aggregates of layers with slit-shape holes, in good agreement with the nitrogen adsorption isotherm.

Brunauer–Emmett–Teller (BET) specific surface area measurements show a large increase in surface area from about 60 m^2^ g^−1^ in the untreated MXene to above 290 m^2^ g^−1^ in the nanoarchitecture (Table S1, ESI), showing an average pore width of ca. 12 nm. In relation to this overall increase in the apparent surface area of the material, the pore volume also underwent a considerable increase of more than 3 times with respect to the initial MXene.

In addition to the increase in specific surface area and porosity, and thus adsorption capacity, the nanoarchitectures built here should show an effective light-to-heat conversion ability, more commonly known as photothermal conversion, provided by the presence of MXene carbide. This property shown on MXene is an emerging topic that is attracting increased interest, focusing on important applications such as steam generation, water desalination, and cancer therapy [9,32]. Photothermal conversion is also remarkable in nanocomposite materials such as those containing biopolymers (e.g., nanocellulose and chitin) assembled with MXenes, which incorporate thermoelectric properties useful in biomedicine [8] and even in amazing behaviors such as controllable light-driven photothermal properties inherent to their 2D carbide component [10].

**Figure 4:**
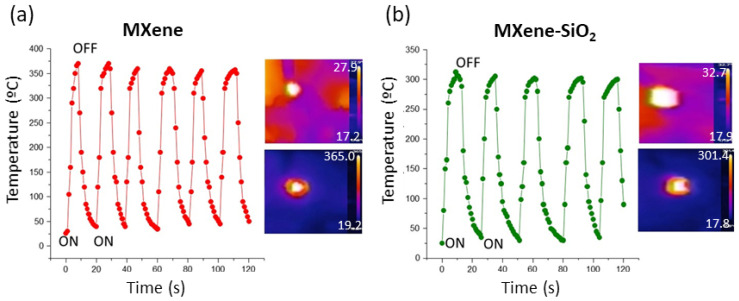
Photothermal cycles upon near infrared irradiation on MXene (**a**) and MXene-SiO_2_ (**b**) and maximum and minimum temperature pictures.

Cyclic on–off laser irradiation at regular time intervals shows a concomitant increase in temperature (Figure 4), demonstrating excellent repetitive photothermal response activity over long periods of time. We have found that this activity is maintained by storing the samples at atmosphere without special storage precautions at least one year after preparation, which indicates a high stability of these MXene materials with respect to their photothermal response.

The multifunctionality of the MXene-SiO_2_ materials, i.e., their high porosity, together with the properties conferred by the inclusion of the MXene, are of interest in terms of their potential photoactivity and electrical conductivity. These properties are theoretically promising for potential applications such as, e.g., the active phase of sensors, porous electrodes, heterogeneous catalysis, etc. [10,11].

Interestingly, the MXene-SiO_2_ nanoarchitectures prepared here retain the characteristic photothermal properties of the MXene component and add the surface properties of the associated porous silica. In this regard, we have shown that the adsorption of organic molecules on MXene-SiO_2_ can be further transformed following a photothermal activation based on NIR laser irradiation. Thus, in a preliminary approach consisting in the adsorption of 2,3-dimethylbutane-2,3-diol (pinacol) on MXene-SiO_2_ after acid doping and NIR laser irradiation, a fast and efficient rearrangement reaction to 3,3-dimethylbutan-2-one (pinacolone) was observed in a similar way to when it occurs in the liquid phase or when it is adsorbed on porous solids [33,34]. As is well known, the rearrangement reactions of 1,2-diols catalyzed by acids or Lewis solids represent a classical model in organic chemistry [33], wherein thermal activation has traditionally been achieved either by heating in a conventional oven or under microwave irradiation [34]. In the present case, a very short irradiation time (10 min) with an NIR laser attenuated to heat at 80–100 °C on H_2_SO_4_-doped MXene-SiO_2_ containing adsorbed pinacol produces its rapid transformation into pinacolone, as detected by the 2,4-dinitrophenylhydrazine test with the formation of orange-colored 2,4-dinitrophenylhydrazone [35].

## 4. Conclusions

Here, we present a new approach for the development of photothermally responsive porous nanoarchitectures by assembling Ti_3_C_2_T_x_ MXene, exfoliated by CTAB intercalation, with silica generated by the hydrolysis of an alkoxysilane (e.g., TMOS). These initial results are a proof of concept that warrants great interest in pursuing this research further. Thus, future developments could be envisaged, incorporating different types of MXenes and various porous systems capable of assembling at the nanoscale to preserve their inherent photoactive response with their adsorption properties and potential reactivity, including catalytic transformations.

Moreover, the presence of porous silica offers the possibility of additional post-synthesis functionalization, for instance, by anchoring functional groups via silanization or by the inclusion of metal and metal-oxide nanoparticles within the large pores generated in these nanoarchitectures. These possibilities open up much broader perspectives for the design and preparation of new multifunctional materials for many different applications, applicable, for instance, to the design of new heterogeneous catalysts or as the active phase of sensor devices. On the other hand, although the current MXene-SiO_2_ nanoarchitectures have a very low electrical conductivity (practically undetectable, due to the elevated content in insulating silica), they might be increased in future works by incorporating highly conductive carbon nanoparticles (e.g., MWCNT and GNP), in these nanoarchitectures with the aim of drastically improving these properties, as has already been achieved in related systems.

Further research is needed to ratify and extend the preparation processes of this type of innovative materials based on MXene-derived nanoarchitectured porous systems, as initially described here, where a simple model reaction of thermal activation has been selected. This study represents only an initial example that opens broad application possibilities to different catalytic and non-catalytic organic reactions amenable to photothermal activation.

## Data Availability

Data are contained within the article.

## Supplementary Materials

The following supporting information can be downloaded at https://www.mdpi.com/article/10.3390/ma17174273/s1, Figure S1. Energy-dispersive X-ray spectroscopy (EDX) of MXene; Figure S2. Energy-dispersive X-ray spectroscopy (EDX) of MXene-SiO_2_; Figure S3. FE-SEM image of Ti_3_AlC_2_ (MAX); Figure S4. ATR spectra of MXene-SiO_2_ and MXene-CTAB samples. Table S1. Textural characteristics of MXene and MXene-SiO_2_ samples.
