# Supplementary material for: Silica–Ti3C2Tx MXene Nanoarchitectures with Simultaneous Adsorption and Photothermal Properties
Source: Materials (Basel). 2024 Aug 29;17(17):4273. doi: 10.3390/ma17174273 (PMC11396753; doi:10.3390/ma17174273)
Supplement: Supplementary file 1 [file materials-17-04273-s001.zip › materials-3063122-supplementary.pdf]

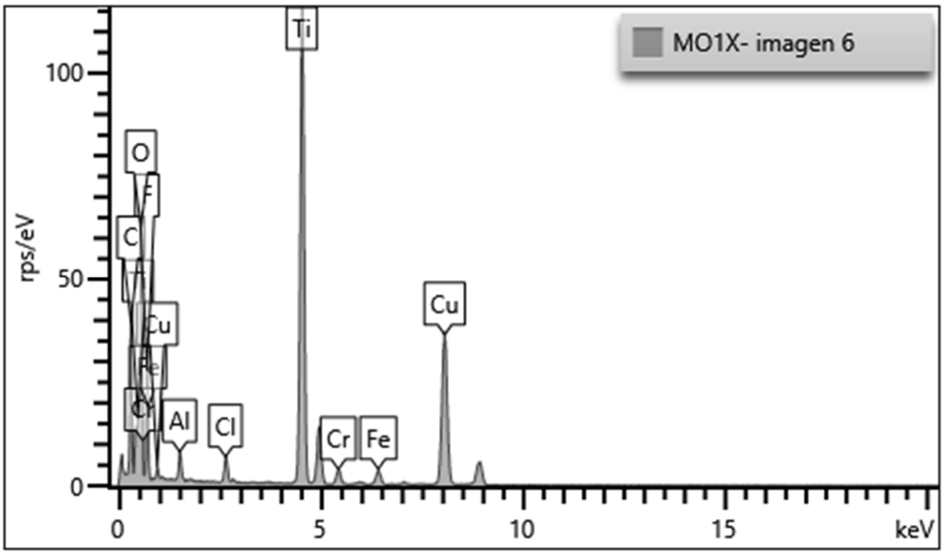

Elemento	Tipo de línea	Factor k	Tipo de factor k	Corrección de absorción	%peso	Sigma % peso	% atómico
O	Serie K	1,94	Teórico	1	25,14	0,26	44,95
F	Serie K	1,662	Teórico	1	9,77	0,17	14,71
Al	Serie K	1,039	Teórico	1	2,06	0,07	2,18
Cl	Serie K	1,026	Teórico	1	2,36	0,07	1,91
Ti	Serie K	1,128	Teórico	1	60,68	0,26	36,25
Total:					100		100

Figure S1. Energy-dispersive X-ray spectroscopy (EDX) of MXene

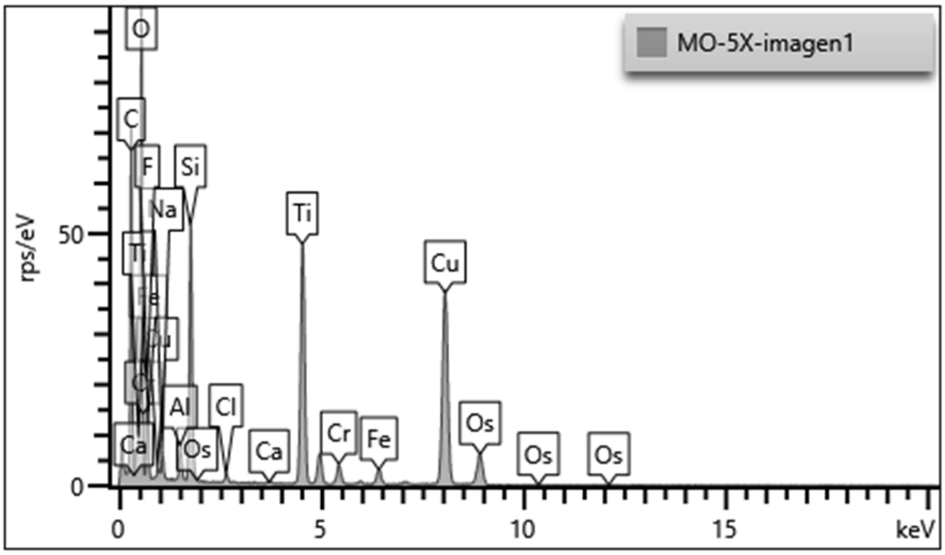

Elemento	Tipo de línea	Factor k	Tipo de factor k	Corrección de absorción	%peso	Sigma % peso	% atómico
O	Serie K	1,94	Teórico	1	42,03	0,26	59,51
F	Serie K	1,662	Teórico	1	7,78	0,17	9,27
Na	Serie K	1,169	Teórico	1	1,58	0,07	1,56
Al	Serie K	1,039	Teórico	1	2,15	0,07	1,8
Si	Serie K	1	Teórico	1	17,27	0,16	13,93
Cl	Serie K	1,026	Teórico	1	0,66	0,05	0,42
Ca	Serie K	1,005	Teórico	1	0,16	0,04	0,09
Ti	Serie K	1,128	Teórico	1	28,38	0,19	13,42
Total:					100		100

Figure S2. Energy-dispersive X-ray spectroscopy (EDX) of MXene- SiO_2

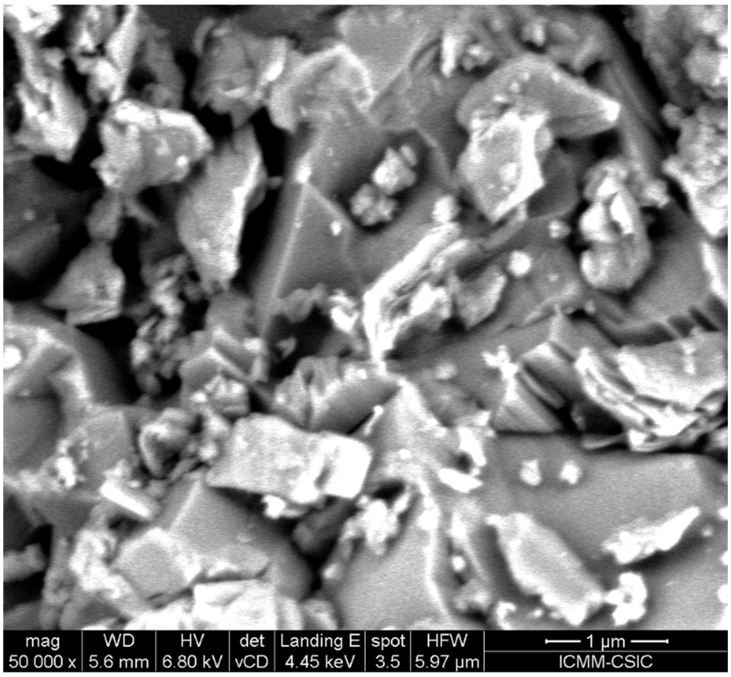

Figure S3. FE-SEM image of Ti_3AlC_2 (MAX)

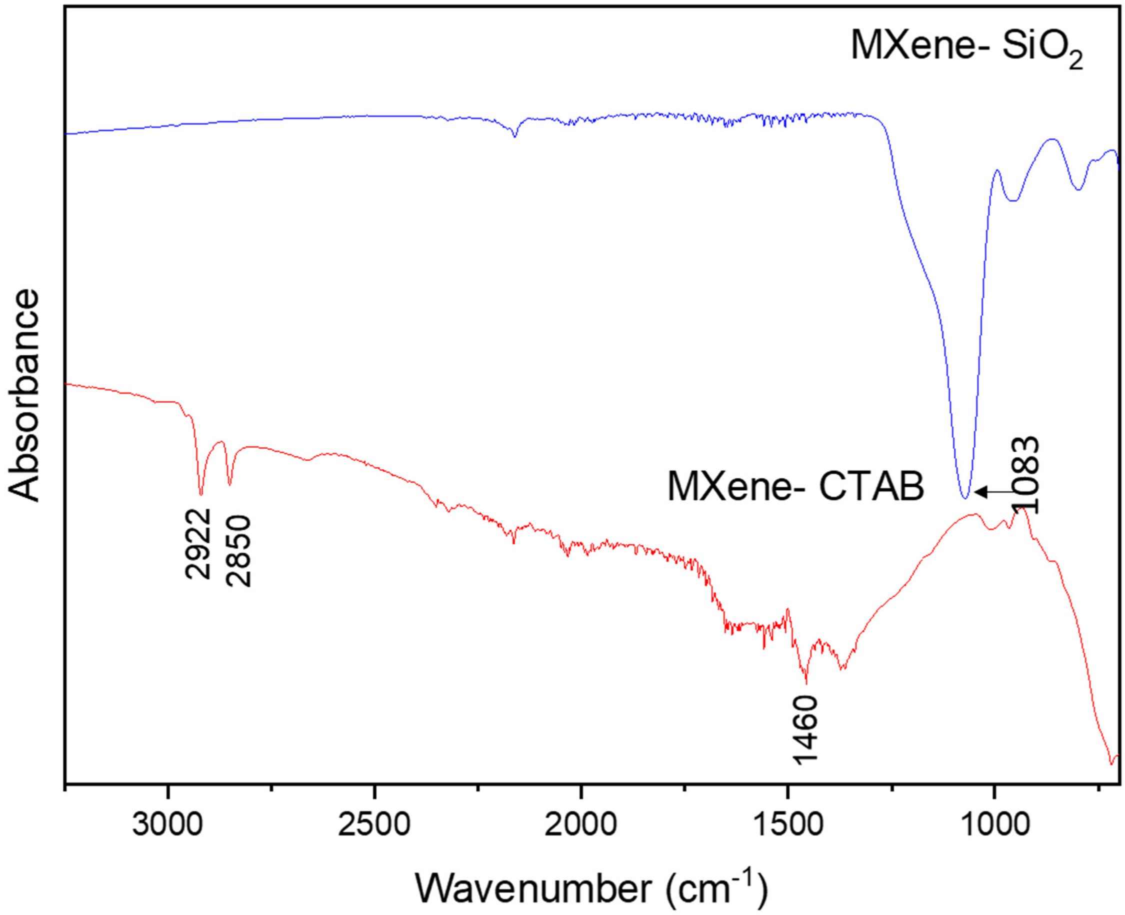

Figure S4. ATR spectra of MXene- SiO_2 and MXene- CTAB samples.

Table S1. Textural characteristics of MXene and MXene- SiO_2 samples.

**Samples**	**S_{BET} (m^2 g^{-1})**	**V_{tot} (cm^3 g^{-1})**	**V_{micro} (cm^3 g^{-1})**	**P_w (nm)**
MXene	59	0,282	0,004	24,6
MXene-SiO2	291	0,933	0,012	12,7

S_{BET} = specific surface area; V_{Total} = total pore volume; and V_{Micro} = micropore volume; P_w =average pore width.
